# Temporal variation of eukaryotic community structures in UASB reactor treating domestic sewage as revealed by 18S rRNA gene sequencing

**DOI:** 10.1038/s41598-019-49290-y

**Published:** 2019-09-04

**Authors:** Yuga Hirakata, Masashi Hatamoto, Mamoru Oshiki, Takahiro Watari, Kyohei Kuroda, Nobuo Araki, Takashi Yamaguchi

**Affiliations:** 1 Department of Science of Technology Innovation, Nagaoka University of Technology, 1603-1 Kamitomioka, Nagaoka, Niigata 940-2188 Japan; 2 Department of Civil and Environmental Engineering, Nagaoka University of Technology, 1603-1 Kamitomioka, Nagaoka, Niigata 940-2188 Japan; 3 Department of Civil Engineering, National institute of Technology, Nagaoka College, 888 Nishikatakaimachi, Nagaoka, Niigata 940-0834 Japan; 4 Department of Chemical Science and Engineering, National Institute of Technology, Miyakonojo College, 473-1 Yoshio-cho, Miyakonojo, Miyazaki 885-8567 Japan

**Keywords:** Next-generation sequencing, Microbial ecology

## Abstract

Eukaryotes are important components of ecosystems in wastewater treatment processes. However, little is known about eukaryotic community in anaerobic wastewater treatment systems. In this study, eukaryotic communities in an up flow anaerobic sludge blanket (UASB) reactor treating domestic sewage during two years of operation were investigated using V4 and V9 regions of 18S rRNA gene for amplicon sequencing. In addition, activated sludge and influent sewage samples were also analyzed and used as the references for aerobic eukaryotic community to characterize anaerobic eukaryotes. The amplicon sequence V4 and V9 libraries detected different taxonomic groups, especially from the UASB samples, suggesting that commonly used V4 and V9 primer pairs could produce a bias for eukaryotic communities analysis. Eukaryotic community structures in the UASB reactor were influenced by the immigration of eukaryotes via influent sewage but were clearly different from the influent sewage and activated sludge. Multivariate statistics indicated that protist genera *Cyclidium*, *Platyophrya* and *Subulatomonas* correlated with chemical oxygen demand and suspended solid concentration, and could be used as bioindicators of treatment performance. Uncultured eukaryotes groups were dominant in the UASB reactor, and their physiological roles need to be examined to understand their contributions to anaerobic processes in future studies.

## Introduction

Microbial eukaryotes play important roles in wastewater treatment systems. In particular, bacterivory by protists contributes to the reduction of sludge production, the improvement of sludge sedimentation, and the quality of effluent water^1–3^. Additionally, some fungi, for example phylum Ascomycota, are also known to contribute to denitrification and cellulose degradation^4,5^. Eukaryotic communities involved in aerobic wastewater treatment processes have been widely studied, so their community compositions and populations are used as biological indicators of these processes^6,7^.

Eukaryotes have been commonly identified morphologically by microscopic observation. However, identifying protists by microscopic observation is prone to error because some species are fast moving and indiscernibly small^8^. Furthermore, most fungi are difficult to identify by microscopic observation. Recently, instead of by microscopic observation, molecular biological techniques such as clone libraries^9^, quantitative real-time PCR^10^, fluorescence *in situ* hybridization^11^, and high-throughput sequencing techniques^12^ have been applied to analyze a greater diversity of eukaryotes in wastewater treatment systems and other enrivonments (e.g. lake and marine). Among them, 18S rRNA gene amplicon sequencing using high-throughput sequencing techniques is reported as an effective and sensitive method for investigating eukaryotic diversity^13^.

High-throughput sequencing provides a very large number of reads though only short sequences are generated^14^. Thus, current methods for high-throughput sequencing of eukaryotic diversity studies rely on sequencing of variable regions of the 18S rRNA gene. The 18S rRNA genes of eukaryotes have nine hypervariable regions (V1 to V9), which can be used for species identification. A number of recent studies have used different variable regions within the 18S rRNA gene for amplification, including V1-V2^15^, V3^16^, V4, and V9 regions^17^. In particular, both V4 and V9 regions have been used to describe the diversity and variation of eukaryotes found in aerobic wastewater treatment systems^18,19^. Previous studies reported that the molecular markers have different characteristics such as V4 region provide more depth and unique reads and V9 region provide wider coverage of higher taxonomic groups.

In contrast to aerobic eukaryotes, limited information is available on the eukaryotic community structures found in anaerobic wastewater treatment systems. Microbial eukaryotes such as protists and fungi also exist and contribute to the degradation of organic matter in anaerobic wastewater treatment systems^20,21^. A few studies have reported that some protists of phylum Ciliophora positively correlated with the removal of organic matter in anaerobic digesters^22,23^. Therefore, anaerobic eukaryotic communities, especially protists, are expected to change in response to environmental conditions and may be used as indicators of operational conditions, as with aerobic eukaryotes. However, most species of anaerobic eukaryotes have not been extensively investigated, and the relationships between changing eukaryotic community structures and treatment performance in anaerobic wastewater treatment systems remain unclear. In addition, although 18S rRNA gene amplicon sequencing has been applied to investigate eukaryotic communities in anaerobic environments such as marine^17^ and rumen environments^24^, few studies have used this method for anaerobic wastewater treatment systems^25^. Furthermore, there are problems derived from using molecular methods, which cannot determine whether the detected eukaryotes are anaerobic or not, except for known species. Indeed, our previous study could not identify anaerobic eukaryotes by just 18S rRNA gene sequencing because some eukaryotic taxonomic groups include both anaerobic and aerobic organisms^26,27^. In particular, microbial communities in anaerobic wastewater treatment systems may be influenced by the immigration of aerobic microorganisms via wastewater. To characterize the anaerobic eukaryotes involved in wastewater treatment, this issue must be considered.

The primary objective of this study was to investigate eukaryotic community structures in an anaerobic wastewater treatment system. For this purpose, we analyzed eukaryotic communities in an up flow anaerobic sludge blanket (UASB) reactor treating domestic sewage over a two-year operational period. We used 18S rRNA gene amplicon sequencing using two primer pairs, targeting the V4 and V9 regions. Eukaryotic communities in aerobic wastewater treatment systems (i.e., activated sludge) and influent sewage were also analyzed and used as the references for aerobic eukaryotic species to characterize anaerobic eukaryotes. In addition, multivariate statistics were applied to elucidate any correlation between eukaryotic communities and the operational conditions of the UASB reactor using the retrieved anaerobic eukaryotic sequences.

## Results

### Overall eukaryotic communities determined by V4 and V9 regions of 18S rRNA gene sequencing

**Figure 1:**
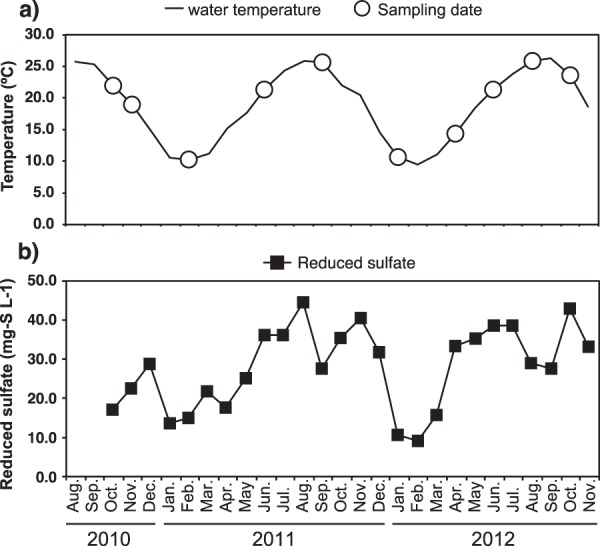
Time courses of (**a**) water temperature and (**b**) reduced sulfate (sulfide) of the UASB reactor and sampling date of sludge samples.

**Figure 2:**
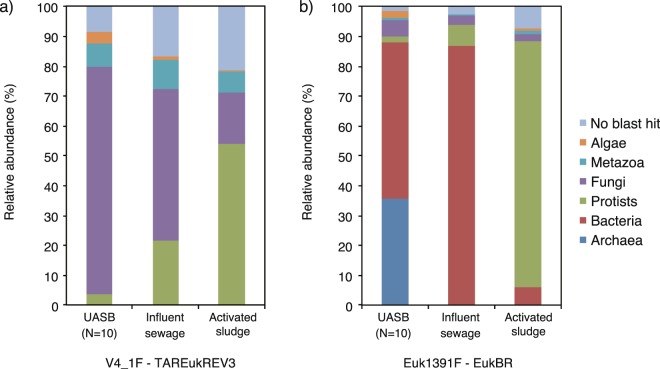
Relative abundance of (**a**) V4 and (**b**) V9 region amplicons assigned to kingdom or domain level in the UASB reactor (N = 10), influent sewage, and activated sludge. Sequence reads that are not classified into any known group were labeled as “No blast hit.”

In this study, eukaryotic community structures were analyzed for 10 samples from a UASB reactor (Fig. 1a), influent sewage, and activated sludge, based on 18S rRNA gene sequencing. A total of 180,678 sequences of the V4 region amplicon library and 340,054 sequences of the V9 region amplicon library were obtained (Supplementary Table S1). The phylogenetic affiliations of all sequences in each ecosystem were classified as archaea, bacteria, protist, fungi, metazoa, and algae at the kingdom or domain level (Fig. 2). The results using the V4 region-specific primer pair showed that the dominant group was fungi in the UASB reactor and influent sewage, whereas protists were dominant in activated sludge. The relative abundances of fungi were 76.1% and 50.8% of the total number of sequences in the UASB reactor and influent sewage, respectively. The relative abundance of fungi in the UASB was continuously high throughout the year (Supplementary Fig. S1a). In contrast, the relative protist abundance was very low in the UASB reactor, accounting for 3.8% of all sequences. Additionally, the relative abundance of algae and metazoa were detected as 0.3–3.9% and 7.1–9.8%, respectively.

However, the results using the V9 region-specific primer pair showed that not only eukaryotic but also a large portion of prokaryotic sequences also detected from the UASB reactor and influent sewage (Fig. 2, Supplementary Tables S2 and S3). The bacteria and archaea in the UASB reactor were accounted for 52.2% and 35.6% of total number of sequence, respectively. On the other hand, protists were dominant in activated sludge samples, where the relative abundance of bacteria was low.

To evaluate eukaryotic communities, sequences classified as archaea and bacteria were excluded from the rest of the analyses. Thus, in the present study, a total of 169,385 and 45,510 eukaryotic sequence reads were generated from the V4 and V9 amplicon libraries, respectively. The alpha diversity was calculated using the lowest sample sizes of eukaryotic sequences for comparison of each amplicon library (Supplementary Table S2). The values of species richness estimates, observed species, Chao1, and ACE were higher in the V4 amplicon library than in the V9 amplicon library. In both amplicon libraries, these values were greater in the UASB reactor than in activated sludge.

### Protist community structures

**Figure 3:**
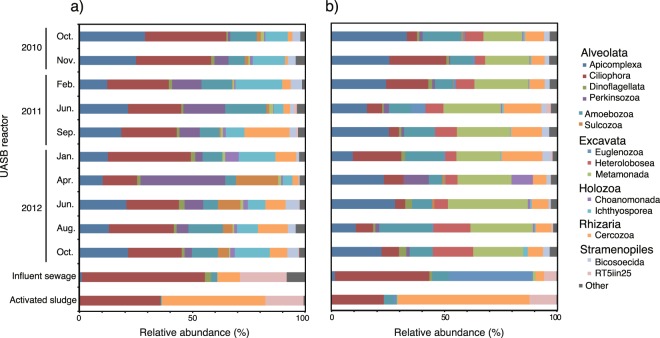
Relative abundance of (**a**) V4 and (**b**) V9 region amplicons assigned to protist phylum level in all samples from the UASB reactor, influent sewage, and activated sludge.

Taxonomic classification of the protist community structures analyzed by V4 and V9 region-specific primer pairs were compared at the phylum level (Fig. 3). In the present study, a total of 3,204 OTUs and 691 OTUs of V4 and V9 amplicon libraries, respectively, were identified (Supplementary Table S4). In the V4 amplicon library, the dominant heterotrophic protist groups in the UASB reactor were phyla Ciliophora and Amoebozoa, with average relative abundances of 27.2% and 10.6%, respectively. Phyla Apicomplexa, Ichthyosporea, and Perkinsozoa, which are known as parasitic protists^28–30^, were also detected in the UASB reactor at average relative abundances of 18.6%, 11.0%, and 10.6%, respectively. The heterotrophic protists identified included phyla Cercozoa, Sulcozoa, Bicosoecida, Choanomonada, Dinoflagellata, and Metamonada (>1% on average).

In the V9 amplicon library from the UASB reactor, phyla Metamonada, Apicomplexa, Amoebozoa, and Ciliophora were dominant, displaying average relative abundances of 23.9%, 21.8%, 12.5%, and 11.0%, respectively. Phyla Ciliophora, Ichthyosporea, Perkinsozoa, and Sulcozoa were found at lower levels in the V9 amplicon library than in the V4 amplicon library. In contrast, phyla Metamonada, Heterolobosea, and Euglenozoa were more abundant in the V9 amplicon library. Although changes in community composition at phylum level were observed in the UASB reactor over the two years, the effect of seasonal changes on protist communities (e.g., winter and summer) was unclear in both amplicon libraries. The alpha diversity indexes of protist sequences includes observed species, Chao1, and ACE had also changed regardless of seasonality in the UASB reactor throughout the two years, while simpson and shannon indexes were not significantly different (Supplementary Table S4). This suggested that protist community could be affected by operational condition than seasonality.

In the V4 amplicon library from influent sewage, the dominant groups were phyla Ciliophora and Cercozoa and the RT5iin25 group, with relative abundances of 54.5%, 9.9%, and 20.5%, respectively. Phyla Ciliophora and Euglenozoa were dominant in the V9 amplicon library from influent sewage, with relative abundances of 41.9% and 37.2%, respectively. In the V9 amplicon library from the UASB reactor and influent sewage, Excavata groups were detected as being more dominant than in the V4 amplicon library. The major groups in activated sludge were similar in both V4 and V9 amplicon libraries, where phyla Ciliophora, Cercozoa, and RT5iin25 groups were dominant.

### Temporal variation in anaerobic protist communities

To determine the factors that influence temporal changes in protist communities in UASB reactors, multivariate statistical analysis was conducted to assess correlation between the major anaerobic protists and environmental parameters. For the analysis, the V4 amplicon libraries were used due to the primer pair’s specificity for eukaryotic sequences. Anaerobic protist genera in the UASB reactor were identified by using eukaryotic sequence obtained from activated sludge and influent sewage as reference of aerobic protist (Supplementary Fig. S2). In the UASB reactor, some protists were from known aerobic genera such as *Epistylis*, *Telotrochidium*, *Tetrahymena*, *Vorticella*, within phylum Ciliophora^31^; *Phalansterium* and *Saccamoeba* within phylum Amoebozoa^32^; *Cercomonas*, *Heteromita*, and *Rhogostoma* within phylum Cercozoa^33,34^; and *Protoperidinium* within phylum Dinoflagellata^35^ which were frequently detected. These protist genera were also detected in influent sewage and activated sewage. The parasitic protist *Cryptosporidium* (phylum Apicomplexa) was detected in both influent sewage and in the UASB reactor. Although genera *Acanthamoeba* and *Tracheloraphis* that could prey other protist cell^36,37^ were also detected in the UASB reactor, these protist and other protist genera were not correlated. These common protist genera in both influent sewage and the UASB reactor accounted for 25.8% of the total protist sequences from the UASB reactor. In contrast, the general anaerobic protists of *Metopus* (phylum Ciliophora) and *Trimastix* (phylum Metamonada)^38,39^ were detected only in the UASB reactor. Additionally, protist genera belonging to phyla Sulcozoa, Bicosoecida, Choanozoa, and Metamonada were exclusively found in the UASB reactor.

**Figure 4:**
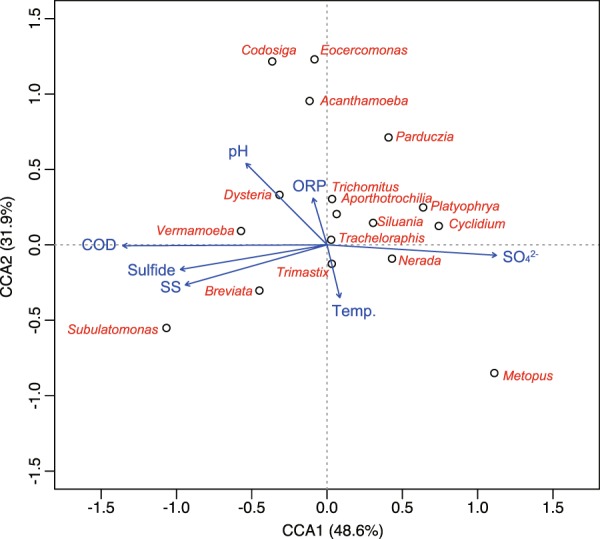
Canonical correspondence analysis based on treatment performance and on the 17 unique protist genera of the UASB reactor having a mean relative abundance per sample of 0.3% or above.

The correlations between anaerobic protist genera and treatment performance in the UASB reactor were examined using CCA (Fig. 4). CCA includes the anaerobic protist genera that were specific to the UASB reactor (Supplementary Fig. S2) and the environmental parameters (Supplementary Table S5). As shown by CCA, genus *Subulatomonas* (phylum Sulcozoa) positively correlated with effluent in COD, SS, and sulfide, whereas *Platyophrya* and *Cyclidium* (phylum Ciliophora) showed negative correlations. Furthermore, comparisons of variation of operational condition and alpha diversity over two years showed that value of estimated species, Chao1 and ACE of protist in V4 amplicon library seemed high when COD concentration of UASB effluent was low (Supplementary Tables S4 and S5). These results also supported that some protist population changed in response to environmental conditions. There were no protist genera that were correlated clearly with water temperature. The one-way analysis of variance (ANOVA) was also performed to analyze difference of relative abundance of protist genera at different season; summer (23.6–26.4 °C); winter (10.3–14.5 °C); spring and fall (18.7–21.6 °C). However, no significant differences were found (data not shown). The principal coordinate analysis (PCoA) showed that protist community structures were not influenced by temperature and reduced sulfate (Supplementary Fig. S3).

### Fungal community structures

**Figure 5:**
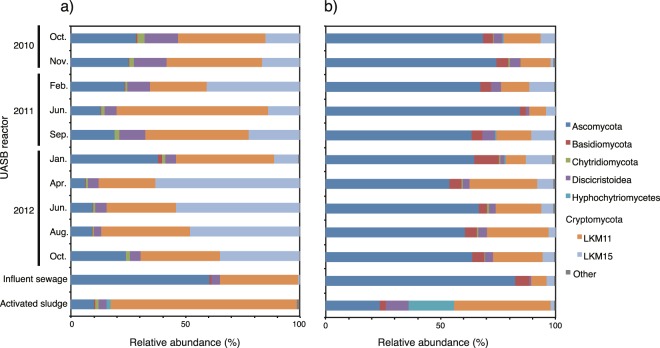
Relative abundance of (**a**) V4 and (**b**) V9 region amplicons assigned to fungi phylum level in all samples of UASB reactor, influent sewage, and activated sludge.

Taxonomic classification of the fungal community structures analyzed by V4 and V9 region-specific primer pairs were compared at the phylum level (Fig. 5). In total, 15,109 and 527 OTUs were identified from fungal amplicons using V4 and V9, respectively (Supplementary Table S6). These sequence reads were classified as phyla Ascomycota, Basidiomycota, Chytridiomycota, Discicristoidea, Hyphochytriomycetes, and uncultured LKM11 and LKM15 groups in phylum Cryptomycota. The relative abundance of the fungi was 17.4–85.8% of the V4 amplicon library in all sequences of each sample. In the V4 amplicon library from the UASB reactor, the dominant fungi were the LKM11 and LKM15 groups, with average relative abundances of 38.8% and 31.7% of all fungal sequences, respectively. In the activated sludge, the LKM11 group was most abundant, accounting for 81.5%. Phylum Ascomycota and the LKM11 group were also detected in the influent sewage, with relative abundances of 60.5% and 34.2%, respectively. In comparison with the V4 amplicon library, the V9 amplicon library showed a drastically different composition (Fig. 5), and phylum Ascomycota was dominantly detected in all samples. Within sequences belonging to phylum Ascomycota in the V9 amplicon library from the UASB reactor, genus *Candida* accounted for 84.7%. The community structures and alpha diversity of fungi had changed regardless of seasonality and reduced sulfate in the UASB reactor throughout the two years as with protist community (Supplementary Table S6). The PCoA results supported that fungi community structures were not influenced by temperature and reduced sulfate (Supplementary Fig. S4).

Phylogenetic analyses of predominant OTUs belonging to uncultured LKM11 and LKM15 groups in phylum Cryptomycota were performed using 18S rRNA gene sequences from the V4 amplicon library (Supplementary Fig. S5). The abundance of OTUs belonging to the LKM11 group varies between samples. The OTU denovo13315 was detected only in the UASB reactor, with an average relative abundance of 8.1%. The OTU denovo4805 was most dominant in activated sludge, at a relative abundance of 49.5%, and was hardly detected in other samples. These results suggested that these two species, OTU denovo13315 and denovo4805, could live in the UASB reactor or activated sludge. Additionally, the OTU denovo9985 was dominant in influent sewage. Other OTUs denovo15978, 18515, 20531, and 22061 belonged to the LKM11 group and were detected in all samples. By contrast, OTUs belonging to the LKM15 group were detected only in the UASB reactors. The OTU denovo23550 was the most dominant, accounting for 97.6% of all OTUs belonging to the LKM15 group.

## Discussion

In this study, the eukaryotic community structures in a UASB reactor fed with domestic sewage were investigated by amplicon sequencing of the V4 and V9 regions of 18S rRNA gene. Eukaryotic communities involved in anaerobic wastewater treatment systems have remained poorly understood in comparison to aerobic processes. To the best of our knowledge, this is the first study of eukaryotic communities involved in anaerobic wastewater treatment that were characterized by comparison between the UASB reactor and influent sewage based on 18S rRNA gene sequencing analysis. Additionally, this study evaluated the applicability of V4 and V9 region-specific primer pairs to characterize eukaryotic communities in the UASB reactor via high-throughput sequencing. The V4 and V9 region-specific primer pairs have been used recently to describe the diversity and communities of eukaryotes in several studies as their flanking regions are well conserved^40,41^. The observed species, Chao1, and ACE indexed of eukaryotic communities were higher in the V4 amplicon library than V9 amplicon library (Supplementary Table S2). This could have resulted from the V4 region amplicon being longer than the V9 region amplicon (V4: 341 bp and V9: 102 bp). In addition, the V4 region-specific primer pair of V4_1F and TAReukREV3 could be used to specifically amplify eukaryotic sequences from all samples, making them available for investigations of eukaryotes in anaerobic treatment systems.

The V9 region-specific primer pair of Euk1391F and EukBR amplified eukaryotic and prokaryotic sequences from samples collected from the UASB reactor and influent sewage (Fig. 2, Supplementary Table S2). Although there is possibility that detection of prokaryotes resulted from the combined effects of read errors during PCR and sequencing, PCR chimera formation, a total of 288,723 sequences assigned to prokaryotes passed QC (Supplementary Table S1). The V9 region specific primer amplified relatively short sequences (<500 bp) in comparison to Sanger sequencing based methods like clone library (>1 kb). In theoretically, short read sequences minimize the occurrence of chimera formation^17^. Additionally, contig length of eukaryotes and prokaryotes obtained from V9 amplicon libraries were different for 123 ± 13 bp and 103 ± 17 bp, respectively. Thus, detection of large number of prokaryotic sequences is considered not for PCR chimera formation and read errors. The forward primer of Euk1391F is targeted at highly conserved rRNA gene sequence regions among the three domains, meaning that non-eukaryotic sequences have often been amplified^17^. The dominant prokaryotes detected by the primer pair of Euk1391F and EukBR from the UASB reactor and influent sewage were genera *Pseudomonas*, *Syntrophobacter*, and *Arcobacter* within phylum Proteobacteria, and *Methanomassiliicoccus* and *Methanobacterium* affiliated with phylum Euryarchaeota. These anaerobes and facultative anaerobes that have 0–1 and 2–4 mismatches with Euk1391F and EukBR, respectively (Supplementary Table S3, Fig. S6). Despite previous studies using the same V9 region-specific primer pair for both anoxic and aerobic samples, the relative abundance of prokaryotes was very low in all sequences (<1%)^17,42^. The high detection ratio of prokaryotes in the present study may be due to the microbial community including a high proportion of prokaryotes that have no, or low levels of, mismatches with Euk1391F and EukBR. Those prokaryotes were detected from the UASB reactor samples based on 16S rRNA gene sequencing^43,44^. Therefore V9 region-specific primer pair of Euk1391F and EukBR was not suitable for analysis of eukaryotic communities in the UASB reactor.

Protists and fungi are known to be the dominant eukaryotes in anaerobic environments^45,46^. Nevertheless, not only protists and fungi but also metazoa and algae were detected in the UASB reactor (Fig. 2). In V4 and/or V9 amplicon libraries, phyla Charophyta and Chlorophyta in algae and phyla Nematoda and Arthropoda in metazoa were the dominant groups in the UASB reactor and were also detected in influent sewage (Supplementary Figs S7 and S8). The members of phyla Chlorophyta and Charophyta are known as either photosynthetic or aerobic heterotrophic organisms^47,48^. Some species of phylum Nematoda were often observed in wastewater treatment plants and raw municipal wastewater, at the egg stage of their life cycle^49^. However, phylum Arthropoda that known to be intolerant of anoxic conditions and was therefore probably introduced into the UASB reactor via influent sewage, in which they were also detected. Furthermore, aerobic, parasitic protists and some fungal species were detected in the UASB reactor and influent sewage (Figs 3 and 5; Supplementary Figs S2 and S5); this indicates that the presence of these species in influent sewage affects eukaryotic communities in the UASB reactor. These data suggest that eukaryotic species in influent sewage should be considered during identification of anaerobic eukaryotes.

The result of this study showed that 18S rRNA gene amplicon sequencing could reveal larger numbers of protist species in the UASB reactor and activated sludge than the microscopic observations^20,31^ and clone libraries used in previous studies^23,50^. Although many protist groups that are barely observable microscopically were detected by 18S rRNA gene amplicon sequencing, V4 and V9 amplicon libraries detected different compositions within protist sequences. Consistent with our previous study, phylum Ciliophora, which was dominantly observed microscopically in the UASB reactor^20^, was the most dominant group in V4 amplicon library. However, phylum Metamonada, which was not found in microscopic observations, were more dominant than phylum Ciliophora in the V9 amplicon library from the UASB reactor. In addition, some protist groups that were detected at low levels in the V4 amplicon library were strongly detected in the V9 amplicon library (e.g., phylum Metamonada, Heterolobosea, and Euglenozoa). Previous studies have reported that V4 and V9 region-specific primer sequences preferentially detected different protist groups^17,51^. This may be caused by the different detection biases of each primer pair. These protist groups, especially phylum Metamonada, were the most likely to be overlooked microscopically; therefore, their populations in UASB reactors should be examined in future studies.

The protist community structures in the UASB reactor were distinctly different and composed of a wide range of taxonomic groups, compared with influent sewage and activated sludge (Fig. 3). Phyla Cercozoa, Amoebozoa, and Ciliophora were detected in both samples. These phyla are found frequently in both aerobic and anaerobic environments^26,32,52^. In addition, protist groups including these phyla detected from influent sewage and activated sludge in this study were also detected in previous studies throughout the year^50,53^. This result showed that anaerobic protist species could be retrieved by using these eukaryotic sequences obtained from activated sludge and influent sewage as a reference of aerobic species (Supplementary Fig. S2). Contrary to these, the protist phyla Sulcozoa, Bicosoecida, Choanozoa, and Metamonada were found exclusively in the UASB reactor, and not in the influent sewage. These protists were previously found in many anaerobic environments such as animal gut^54^, anoxic sediment of saline lake^55^, anoxic zone of freshwater lake^56^, and marine environments^57–59^. Thus, these protists are anaerobic and could live in the UASB reactor.

The parasitic protists, such as phyla Ichthyosporea and Perkinsozoa, were detected only in the UASB reactor in both amplicon libraries. Species of phyla Ichthyosporea and Perkinsozoa were previously detected in marine^60^ and freshwater environments^11^ and have free-living stages and cyst stages during their life cycles^29,30^. Although it is unclear whether those organisms occur in free-living or parasitic forms in the UASB reactor, this result suggested that these species could grow in the UASB reactor.

Some correlation was found between certain anaerobic protist genera and the environmental parameters of the UASB reactor (Fig. 4). The genera *Cyclidium* and *Platyophrya* (phylum Ciliophora) were negatively correlated with COD and SS concentrations of effluent, suggesting the importance of these protists as indicators of good treatment performance in UASB reactors. These protist genera are bacterivorous species in anaerobic environments^61,62^ and may contribute to the degradation of particulate organic matter. Notably, a positive correlation between genus *Cyclidium* and COD removal, volatile fatty acid (VFA) concentration, and gas production, have been observed in anaerobic digesters previously^22,23^. In contrast, genus *Subulatomonas* (phylum Sulcozoa) and effluent COD and SS were positively correlated. The genus *Subulatomonas*, isolated from anoxic marine sediment, can grow anaerobically with mixed bacteria as a substrate^63^. Additionally, Xie *et al*.^64^ investigated microbial communities in coastal sediment impacted by oil pollution, reporting that the *Subulatomonas* genus was dominant in sediments containing high concentrations of oil. Therefore, it is possible that these species preferentially grow under high organic matter concentrations and might be considered as indicators of poor treatment performance in the UASB reactors.

The seasonality of most protist genera in the UASB reactor was unclear. However, growth efficiency of protists was influenced by water temperature^65^, while some anaerobic protist species showed increased growth rates at temperatures higher than 20 °C^66^. Thus, the total protist population of the UASB reactor may be different in every season, resulting from water temperature changes. To use protists as biological indicators of the UASB reactor, further studies are required to establish associations between protist diversity, population changes, and environmental parameters in detail.

The effect of reduced sulfate on the eukaryotic community was also not found in the UASB reactor even though sulfide could be an important factor of growth inhibition of microorganism. The reduced sulfate increased during high temperature period, and decreased during low temperature period (Fig. 1). No significant differences of relative abundance of protist genera were found between low reduced sulfate period (10.6–21.8 mg-S L^−1^) and high reduced sulfate period (28.9–42.9 mg-S L^−1^) (data not shown). The inside of the UASB reactor is sulfide-rich environment compared than natural environment such as freshwater lake because sulfide-rich sewage was fed into the UASB reactors for a long time and sulfate reduction was always occurred^20,67,68^. This situation possibly resulted in the selective construction of the sulfide-resistant protist community, thus they were not susceptible to sulfide. This phenomenon could also result in the fungi community in the UASB reactor.

Fungi were the dominant eukaryotes in the UASB reactor (Fig. 2). The dominant fungal groups were different in the V4 and V9 amplicon libraries, likely due to differences in the detection biases of the primer pairs, as discussed above. In the V9 amplicon library from the UASB reactor, the dominant fungus group was genus *Candida* (phyla Ascomycota), which was found in the anaerobic digester and known to grow under anaerobic conditions^11^. On the other hand, the uncultured fungal groups of LKM11 and LKM15 in phylum Cryptomycota were dominantly detected in the V4 amplicon library. Although the uncultured LKM11 group in phylum Cryptomycota was detected in all samples, the dominant OTUs of each environment were different (Supplementary Fig. S5). This could have resulted from the LKM11 group including both aerobic and anaerobic species. The LKM11 group was previously detected in activated sludge treating domestic sewage^50^, anaerobic digester^11^, anoxic sediments^69^, and freshwater lake^70^. Some members of the LKM11 group in freshwater are expected to be parasitic fungi or be involved in the decomposition of detritus^12,71^. In contrast, the LKM15 group was detected only in the UASB reactor, indicating their ability to survive and grow in this environment. Sequences of the LKM15 group were found in anoxic environments such as lake or pond sediments^72^. However, the functions of the LKM11 and LKM15 groups in the sewage treatment process are still largely unknown. Our results showed that some members of the LKM11 and LKM15 groups were independent in the UASB reactor from the influent sewage and may be involved in organic degradation in anaerobic wastewater treatment systems. Their functions should be examined in more detail in future studies.

## Conclusion

In summary, this study revealed the eukaryotic communities existing in a UASB reactor fed with domestic sewage, using the V4 and V9 regions of 18S rRNA for gene amplicon sequencing. For the UASB reactor, the V4 region-specific primer pair specifically amplified eukaryotic sequences, whereas the V9 region-specific primer pair was not suitable for analysis of eukaryotic communities in the UASB reactor because large number of prokaryotes sequences were detected. The eukaryotic community structures in the UASB reactor were influenced by the immigration of eukaryotes via influent sewage, but were clearly different from influent sewage and activated sludge. The changes of protist and fungi community structure in the UASB reactor were not influenced by seasonality. In addition, uncultured eukaryotes such as parasitic protists and LKM11 and LKM15 groups of fungi were exclusively detected in the UASB reactor. The physiological roles of these eukaryotes need to be examined to understand their contributions to anaerobic processes in future studies.

## Materials and Methods

### Sample collection

Sludge samples of 50 mL were collected over two years (October 2010–October 2012, Fig. 1a) from a sampling port 1.278 m above the bottom of the UASB reactor. The reactor had a total volume of 1,178 L, was 4.7 m in height, and was located at a domestic sewage treatment center of Nagaoka City, Japan. The UASB reactor was operated without temperature control. To activate the microorganisms responsible for sulfur redox cycles, the system was fed with raw sewage that was supplemented with 50–150 mg-S L^−1^ sodium sulfate. Additional details on the UASB reactor have been previously described^68^. To classify the anaerobic and aerobic eukaryotes species, activated sludge and influent sewage were collected from the same domestic sewage treatment center in February 2017. The collected samples were concentrated by centrifugation at 12,000 rpm and removed supernatant, then immediately stored at −20 °C for 4–6 years until DNA extraction was performed.

### Measurement of environmental parameters

The water temperature and the pH were measured using a pH meter (HM-20P; TOA DKK, Tokyo, Japan). The oxidation-reduction potential (ORP) were measured using an ORP meter (RM-20P; TOA DKK). The chemical oxygen demand (COD) concentration was determined using a HACH water quality analyzer (DR2500; HACH, Loveland, CO, USA). The suspended solid (SS) concentration was also measured using a glass fiber filter (0.4 μm, GB140; Advantec, Tokyo, Japan). The sulfate concentrations were determined by a high-performance liquid chromatography (HPLC) system (LC 20-ADsp; Shimadzu, Kyoto, Japan). The sulfide concentration was measured according to the standard methods published by the Japan Sewage Works Association^73^.

### DNA extraction, PCR amplification, and 18S rRNA gene sequencing

Genomic DNA was extracted from the collected samples using a FastDNA SPIN Kit for Soil (MP Biomedicals, Carlsbad, CA, USA), according to the manufacturer’s protocol. The DNA concentration was determined using a NanoDrop Spectrophotometer ND-1000 (Thermo Fisher Scientific, Waltham, MA, USA). Amplifications of V4 and V9 regions of 18S rRNA genes were performed using eukaryote-specific primer pairs of V4_1F (5′-CCAGCASCYGCGGTAATWCC-3′) - TAReukREV3 (5′-ACTTTCGTTCTTGATYRA-3′) and Euk1391F (5′-GTACACACCGCCCGTC-3′) - EukBR (5′- TGATCCTTCTGCAGGTTCACCTAC-3′), respectively^17,74^. The adapters for Illumina MiSeq sequencing were attached for each primer according to previous study^75^. Premix Ex Taq Hot Start Version (TaKaRa Bio Inc., Shiga, Japan) was used for PCR amplification. The following were the conditions of PCR amplification. For amplification of V4 region, 5 min at 94 °C; 15 cycles of 30 s at 94 °C, 45 s at 53 °C, and 1 min at 72 °C; 20 cycles of 30 s at 94 °C, 45 s at 48 °C, and 1 min at 72 °C; with a final extension step of 10 min at 72 °C. For amplification of V9 region, 5 min at 94 °C; 30 cycles of 30 s at 94 °C, 30 s at 57 °C, and 1 min at 72 °C; with a final extension step of 10 min at 72 °C. The amplicon was purified using an Agencourt AMPure XP Kit (Beckman Coulter, Brea, CA, USA) and concentrations were measured using a BioAnalyzer DNA 1000 (Agilent Technologies, Santa Clara, CA, USA). 18S rRNA gene sequencing was conducted using a MiSeq Reagent Kit v2 nano and a MiSeq system (Illumina, San Diego, CA, USA).

### Data analysis

Sequence reads were processed using Quantitative Insights Into Microbial Ecology (QIIME) version 1.9.0^74^. Sequence reads with low quality scores (Phred quality score ≤30) were eliminated using the Trimmomatic v0.33 program; specifying a sliding window of 4 with average Phred quality of 30 and 60 as the minimum read length to be conserved for quality control^76^. Paired-end sequence reads were then assembled using the paired-end assembler within the Illumina sequence software package (PANDAseq), and at least 20 bp overlapping region was retained^77^. Putative chimeric sequences were detected and removed using UCHIME software^78^. Operational taxonomic units (OTUs) clustering at 97% sequence identity was conducted with the de novo strategy using the UCLUST algorithm^79^. Taxonomic classifications were determined using the SILVA database 128^80^ and BLAST searches (https://blast.ncbi.nlm.nih.gov/Blast.cgi) in the National Center for Biotechnology Information (NCBI) database. The OTU that assigned to prokaryotes using SILVA and NCBI database were excluded from multivariate statistics. Alpha diversity index of eukaryotic sequences from each sample was calculated at a subsampling depth of lowest reads from each amplicon library, protist and fungi sequences. The phylogenic tree was constructedin MEGA software using neighbor-joining methods^81^.

After detrended correspondence analysis (DCA) was performed to determine the appropriate type of model for direct gradient analysis, canonical correspondence analysis (CCA) or redundancy analysis (RDA) were performed to investigate correlations between eukaryotic communities and environmental factors using the ‘vegan’ R package^82^. In this study, CCA analysis was used because gradient length was calculated as 3.1. The value is greater than 2, it is suitable for using CCA^83^. A Monte Carlo test was used to check the significance of multivariate analysis using the ‘ade4’ R package^84^. These analyses included the environmental parameters of the UASB reactor, and 17 anaerobic protist genera, representing at least 0.3% mean relative abundance per sample. The change of protist and fungi community structure in the UASB reactor was evaluated by principal coordinates analysis (PCoA) based on Bray-Curtis distance. The difference of individual eukaryotes groups at a different time was determined by one-way analysis of variance (ANOVA) or Welch’s t-tests.

### Nucleotide sequence accession numbers

Sequence data were deposited in the DDBJ nucleotide sequence database under accession numbers DRA007151.

## Supplementary information

Supplementary Information
