# Supplementary material for: Temporal variation of eukaryotic community structures in UASB reactor treating domestic sewage as revealed by 18S rRNA gene sequencing
Source: Sci Rep. 2019 Sep 4;9:12783. doi: 10.1038/s41598-019-49290-y (PMC6726610; doi:10.1038/s41598-019-49290-y)
Supplement: Supplementary file 1 — Supplementary Information [file 41598_2019_49290_MOESM1_ESM.docx]

Supplementary Information

Temporal variation of eukaryotic community structures in UASB reactor treating domestic sewage as revealed by 18S rRNA gene sequencing

Authors: Yuga Hirakata, Masashi Hatamoto, Mamoru Oshiki, Takahiro Watari, Kyohei Kuroda, Nobuo Araki and Takashi Yamaguchi

Supplementary Figure S1. Relative abundance of a) V4 and b) V9 region amplicons assigned to kingdom or domain level in the UASB reactor. Sequence reads that are not classified into any known group were labeled as “No blast hit.”

Supplementary Figure S2. Relative abundance of V4 region amplicons assigned to protist genus level in all samples from the UASB reactor, influent sewage, and activated sludge. Protist genera (represented by the areas of dots) representing at least 0.3% mean relative abundance per sample are shown. The size of each dot indicates the percentage of protist genera within the protist sequences. The gray colored part indicates shared protist genera in the UASB reactor, influent sewage, and activated sludge.

Supplementary Figure S3. Principal coordinates analysis (PCoA) based on Bray-Curtis distance of the protist OTU of the UASB reactor. Data colored by sampling date (A), reduced sulfate (sulfide) level (B), and temperature (C).

Supplementary Figure S4. Principal coordinates analysis (PCoA) based on Bray-Curtis distance of the fungal OTU of the UASB reactor. Data colored by sampling date (A), reduced sulfate (sulfide) level (B), and temperature (C).


Supplementary Figure S5. A phylogenetic tree based on neighbor-joining (NJ) methods and relative abundance of OTUs assigned to uncultured LKM11 and LKM15 groups in phyla Cryptomycota, using the 18S rRNA gene. The 18S rRNA gene of *Diplogaster* sp. (GenBank accession number FJ516756) was used as an out-group (not shown). The OTUs obtained in this study are shown in bold type in the tree. The OTUs (represented by the areas of dots) representing at least 1.0% mean relative abundance per sample are shown. The size of each dot indicates the percentage of OTUs within the fungal sequences.

Supplementary Figure S6. Sequence alignment of V9-specific primer pair and prokaryotic sequences detected in this study. Prokaryotic sequences were obtained from the NCBI GenBank database. Accession numbers of each prokaryotic sequence are shown in parentheses. The outline characters and black characters indicate matches and mismatches in the alignment, respectively.


Supplementary Figure S7. Relative abundance of a) V9 and b) V4 region amplicons assigned to Metazoa phylum level in all samples from the UASB reactor, influent sewage, and activated sludge

Supplementary Figure S8. Relative abundance of a) V4 and b) V9 region amplicons assigned to Algae phylum level in all samples from the UASB reactor, influent sewage, and activated sludge

| Samples | | V4 | | | | |  | V9 | | | | |
| --- | --- | --- | --- | --- | --- | --- | --- | --- | --- | --- | --- | --- |
|  |  | Raw sequences | After QC* | After assembly | non-chmeric | OTUs |  | Raw sequences | After QC* | After assembly | non-chmeric | OTUs |
| 2010 | Oct. | 44873 | 40092 | 23119 | 22742 | 4342 |  | 71112 | 68908 | 62796 | 62735 | 1356 |
|  | Nov. | 35723 | 30305 | 16010 | 15609 | 4141 |  | 54145 | 52851 | 48424 | 48385 | 1357 |
| 2011 | Feb. | 35025 | 30883 | 18351 | 18060 | 3110 |  | 28450 | 27581 | 25431 | 25404 | 1042 |
|  | Jun. | 30551 | 26693 | 16787 | 16537 | 2966 |  | 39604 | 38412 | 35410 | 35400 | 1234 |
|  | Sep. | 37367 | 32203 | 18862 | 18472 | 4095 |  | 35459 | 34113 | 31356 | 31354 | 1138 |
| 2012 | Jan. | 30420 | 24427 | 11761 | 11554 | 2733 |  | 19937 | 19202 | 15448 | 15435 | 1020 |
|  | Apr. | 25133 | 22435 | 14185 | 14020 | 2205 |  | 24792 | 23971 | 19827 | 19822 | 929 |
|  | Jun. | 26277 | 24197 | 15761 | 15583 | 2360 |  | 20766 | 20171 | 16900 | 16893 | 809 |
|  | Aug. | 29707 | 25655 | 13784 | 13604 | 2656 |  | 31287 | 30172 | 24970 | 24957 | 1206 |
|  | Oct. | 39014 | 34249 | 20784 | 20412 | 3840 |  | 38763 | 37618 | 31192 | 31181 | 1078 |
| Influent sewage | | 40959 | 17288 | 7160 | 7075 | 1400 |  | 16695 | 16550 | 15453 | 15429 | 663 |
| Activated sludge | | 54805 | 23916 | 7174 | 7010 | 1377 |  | 17125 | 16497 | 13060 | 13059 | 697 |
| Total | | 429854 | 332343 | 183738 | 180678 | 23908 |  | 398135 | 386046 | 340267 | 340054 | 4772 |
| *QC: quality control | | | | | | | | | | | | |

Supplementary Table S1. Number of raw sequence reads and post QC sequence reads

Supplementary Table S2. SSU rRNA gene data and alpha‐diversity indices of eukaryotic communities resulting from V4 and V9 regions of 18S rRNA gene sequencing

|  |  | Sequence number | | | | |  | Alpha‐diversity indices of eukaryotes* | | | | | |
| --- | --- | --- | --- | --- | --- | --- | --- | --- | --- | --- | --- | --- | --- |
| V9 | | Total reads | Eukaryotes | Archaea | Bacteria | No blast hit |  | Observed species | Simpson | Shannon | Chao1 | ACE | Goods_coverage |
| 2010 | Oct. | 35187 | 4998 | 9395 | 20697 | 97 |  | 161 | 0.94 | 3.81 | 291 | 348 | 0.96 |
|  | Nov. | 62511 | 4132 | 34684 | 23628 | 67 |  | 143 | 0.90 | 3.51 | 247 | 286 | 0.95 |
| 2011 | Feb. | 48796 | 6350 | 23761 | 18571 | 114 |  | 143 | 0.90 | 3.52 | 404 | 364 | 0.96 |
|  | Jun. | 25258 | 6714 | 8636 | 9848 | 60 |  | 132 | 0.73 | 2.76 | 368 | 436 | 0.97 |
|  | Sep. | 31131 | 5444 | 10622 | 14983 | 82 |  | 151 | 0.93 | 3.68 | 298 | 334 | 0.97 |
| 2012 | Jan. | 15746 | 1220 | 2389 | 11560 | 577 |  | 170 | 0.95 | 3.91 | 443 | 473 | 0.87 |
|  | Apr. | 20176 | 1081 | 4994 | 13419 | 682 |  | 159 | 0.96 | 4.07 | 337 | 350 | 0.89 |
|  | Jun. | 17067 | 766 | 5262 | 10621 | 418 |  | 155 | 0.92 | 3.71 | 327 | 389 | 0.86 |
|  | Aug. | 25477 | 1788 | 6794 | 15784 | 1111 |  | 182 | 0.95 | 4.05 | 507 | 542 | 0.91 |
|  | Oct. | 31600 | 1109 | 9395 | 20471 | 917 |  | 157 | 0.92 | 3.82 | 238 | 270 | 0.91 |
| Influent sewage | | 15812 | 1593 | 47 | 13454 | 718 |  | 123 | 0.90 | 3.26 | 286 | 331 | 0.93 |
| Activated sludge | | 14384 | 11765 | 0 | 807 | 1812 |  | 62 | 0.67 | 2.01 | 121 | 119 | 0.99 |
| Total | | 340054 | 46286 | 115750 | 173389 | 4629 |  |  |  |  |  |  |  |
|  | | | | | | | | | | | | | |
|  | | | | | | | | | | | | | |
| V4 | |  |  |  |  |  |  |  |  |  |  |  |  |
| 2010 | Oct. | 22742 | 20940 | 0 | 5 | 1797 |  | 247 | 0.97 | 4.49 | 821 | 988 | 0.85 |
|  | Nov. | 15609 | 14171 | 0 | 3 | 1435 |  | 310 | 0.96 | 4.64 | 1517 | 1448 | 0.79 |
| 2011 | Feb. | 18060 | 16781 | 0 | 7 | 1272 |  | 211 | 0.88 | 3.70 | 888 | 1090 | 0.87 |
|  | Jun. | 16537 | 14685 | 0 | 4 | 1848 |  | 196 | 0.84 | 3.37 | 1243 | 1150 | 0.86 |
|  | Sep. | 18472 | 16460 | 0 | 6 | 2006 |  | 251 | 0.93 | 4.10 | 1298 | 1165 | 0.83 |
| 2012 | Jan. | 11554 | 10089 | 0 | 2 | 1463 |  | 254 | 0.96 | 4.36 | 1279 | 1248 | 0.83 |
|  | Apr. | 14020 | 13146 | 0 | 3 | 871 |  | 149 | 0.69 | 2.58 | 809 | 938 | 0.88 |
|  | Jun. | 15583 | 14633 | 0 | 2 | 948 |  | 170 | 0.76 | 2.92 | 1200 | 1462 | 0.89 |
|  | Aug. | 13604 | 12695 | 0 | 5 | 904 |  | 212 | 0.88 | 3.61 | 1239 | 1615 | 0.85 |
|  | Oct. | 20412 | 18916 | 0 | 5 | 1491 |  | 241 | 0.92 | 4.04 | 1726 | 1383 | 0.86 |
| Influent sewage | | 7075 | 5891 | 0 | 8 | 1176 |  | 199 | 0.90 | 3.75 | 703 | 689 | 0.88 |
| Activated sludge | | 7010 | 5513 | 0 | 3 | 1494 |  | 193 | 0.93 | 3.84 | 742 | 793 | 0.88 |
| Total | | 180678 | 163920 | 0 | 53 | 16705 |  |  |  |  |  |  |  |
| *Calculation at a sampling depth of 700 reads. | | | | | | | | | | | | | |

|  | | | | | |
| --- | --- | --- | --- | --- | --- |
| OTUs* | Accession No. | Closely related species | UASB  (N = 10) | Influent  sewage | Activated sludge |
| denovo1429 | JQ045817 | *Pseudomonas poae* | 0.1% | 29.3% | 0.1% |
| denovo3211 | MG195900 | *Arcobacter suis* | 0.0% | 18.5% | 0.0% |
| denovo2044 | NR_075002 | *Syntrophobacter fumaroxidans* | 14.7% | 0.0% | 0.0% |
| denovo428 | CP005934 | *Methanomassiliicoccus intestinalis* | 12.6% | 0.0% | 0.0% |
| denovo3974 | MG195891 | *Arcobacter bivalviorum* | 0.0% | 8.0% | 0.0% |
| denovo3298 | AF028688 | *Methanobacterium bryantii* | 6.5% | 0.0% | 0.0% |
| denovo913 | NR_028247 | *Methanobacterium subterraneum* | 5.1% | 0.0% | 0.0% |
| denovo3127 | FJ193683 | *Acinetobacter sp.* | 0.1% | 5.0% | 0.1% |
| denovo694 | KT862103 | *Dickeya chrysanthemi* | 0.6% | 4.8% | 0.1% |
| denovo4667 | X97691 | *Pedomicrobium manganicum* | 4.2% | 0.0% | 0.0% |
| * Top 10 OTUs selected from all prokaryotic sequences of the V9 amplicon library among all samples | | | | | |

Supplementary Table. S3. The relative abundances of dominant prokaryotic OTUs in all prokaryotic sequences of the V9 amplicon library

| Samples | | V4 | | | | | | |  | V9 | | | | | | |
| --- | --- | --- | --- | --- | --- | --- | --- | --- | --- | --- | --- | --- | --- | --- | --- | --- |
|  |  | sequences | OTUs | shannon^a^ | simpson^a^ | observed species^a^ | Chao1^a^ | ACE^a^ |  | sequences | OTUs | shannon^b^ | simpson^b^ | observed species^b^ | Chao1^b^ | ACE^b^ |
| 2010 | Oct. | 1106 | 370 | 4.08 | 0.96 | 131 | 519 | 647 |  | 935 | 141.0 | 3.03 | 0.90 | 40 | 79 | 86 |
|  | Nov. | 833 | 378 | 4.41 | 0.98 | 151 | 1120 | 962 |  | 1005 | 144 | 2.66 | 0.87 | 32 | 67 | 68 |
| 2011 | Feb. | 787 | 307 | 4.16 | 0.96 | 139 | 772 | 692 |  | 1444 | 171 | 3.05 | 0.91 | 28 | 57 | 104 |
|  | Jun. | 428 | 211 | 4.23 | 0.96 | 154 | 1268 | 1515 |  | 1016 | 155 | 2.79 | 0.88 | 42 | 96 | 147 |
|  | Sep. | 829 | 395 | 4.64 | 0.98 | 174 | 1150 | 1240 |  | 1089 | 141 | 2.85 | 0.90 | 38 | 66 | 87 |
| 2012 | Jan. | 324 | 204 | 4.87 | 0.99 | 188 | 713 | 891 |  | 177 | 70 | 3.27 | 0.94 | 45 | 123 | 131 |
|  | Apr. | 517 | 201 | 3.69 | 0.90 | 127 | 443 | 706 |  | 239 | 61 | 2.87 | 0.90 | 33 | 52 | 72 |
|  | Jun. | 324 | 156 | 4.45 | 0.98 | 148 | 555 | 559 |  | 132 | 46 | 2.90 | 0.88 | 38 | 147 | 176 |
|  | Aug. | 546 | 283 | 4.51 | 0.97 | 167 | 724 | 835 |  | 362 | 87 | 3.11 | 0.91 | 41 | 104 | 137 |
|  | Oct. | 611 | 285 | 4.53 | 0.98 | 152 | 479 | 583 |  | 200 | 53 | 2.81 | 0.89 | 38 | 173 | 133 |
| Influent sewage | | 1510 | 426 | 4.33 | 0.97 | 138 | 499 | 452 |  | 1080 | 114 | 2.84 | 0.89 | 34 | 97 | 99 |
| Activated sludge | | 3799 | 617 | 3.26 | 0.88 | 93 | 313 | 440 |  | 11285 | 171 | 1.80 | 0.70 | 19 | 45 | 75 |
| Total | | 11614 | 3204 |  |  |  |  |  |  | 18964 | 691 |  |  |  |  |  |
| ^a^ Calculation at a sampling depth of 300 reads, ^b^ Calculation at a sampling depth of 100 reads | | | | | | | | | | | | | | | | |

Supplementary Table S4. Alpha diversity indexes calculated from protist sequences.

Supplementary Table S5. Operational conditions and performance of the UASB reactor treating domestic sewage

| Year | Month | UASB reactor | | |  | Water quality of influent | | | |  | Water quality of effluent | | | |
| --- | --- | --- | --- | --- | --- | --- | --- | --- | --- | --- | --- | --- | --- | --- |
|  |  | Temp. | pH | ORP |  | SO_4_^2−^ | Sulfide | SS | COD |  | SO_4_^2−^ | Sulfide | SS | COD |
|  |  | °C |  | mV |  | mg-S L^−1^ | mg-S L^−1^ | mg L^−1^ | mg L^−1^ |  | mg-S L^−1^ | mg-S L^−1^ | mg L^−1^ | mg L^−1^ |
| 2010 | Oct. | 21.6 | 6.8 | −241 |  | 7.2 | 0.0 | 93.0 | 299.3 |  | 8.1 | 17.0 | 31.7 | 120.1 |
|  | Nov. | 18.7 | 7.0 | −246 |  | 86.4 | 1.3 | 76.4 | 288.8 |  | 78.1 | 21.8 | 27.6 | 144.7 |
| 2011 | Feb. | 10.3 | 7.0 | −182 |  | 109.6 | 1.5 | 92.4 | 317.8 |  | 79.0 | 15.0 | 48.5 | 198.8 |
|  | Jun. | 21.6 | 6.8 | −211 |  | 35.7 | 1.6 | 128.8 | 341.9 |  | 10.0 | 36.1 | 41.6 | 164.5 |
|  | Sep. | 25.6 | 6.8 | −277 |  | 34.2 | 3.5 | 89.2 | 383.0 |  | 4.2 | 31.7 | 38.9 | 145.1 |
| 2012 | Jan. | 10.6 | 7.3 | −175 |  | 54.1 | 2.5 | 71.2 | 314.7 |  | 47.6 | 10.6 | 29.6 | 188.0 |
|  | Apr. | 14.5 | 7.1 | −203 |  | 45.8 | 1.1 | 110.6 | 383.8 |  | 12.6 | 33.4 | 58.5 | 265.0 |
|  | Jun. | 21.4 | 6.9 | −234 |  | 58.1 | 1.6 | 103.1 | 384.3 |  | 2.3 | 38.5 | 38.7 | 238.1 |
|  | Aug. | 26.4 | 6.8 | −280 |  | 37.3 | 2.7 | 98.7 | 372.7 |  | 3.7 | 28.9 | 40.3 | 172.7 |
|  | Oct. | 23.6 | 6.8 | −262 |  | 54.0 | 2.7 | 117.4 | 377.4 |  | 1.4 | 42.9 | 47.4 | 229.6 |

Supplementary Table S6. Alpha diversity indexes calculated from fungi sequences.

| Samples | | V4 | | | | | | |  | V9 | | | | | | |
| --- | --- | --- | --- | --- | --- | --- | --- | --- | --- | --- | --- | --- | --- | --- | --- | --- |
|  |  | sequences | OTUs | shannon^a^ | simpson^a^ | observed species^a^ | Chao1^a^ | ACE^a^ |  | sequences | OTUs | shannon ^b^ | simpson ^b^ | observed species ^b^ | Chao1 ^b^ | ACE ^b^ |
| 2010 | Oct. | 17024 | 2729 | 4.35 | 0.95 | 308 | 1434 | 1247 |  | 2444 | 169 | 2.48 | 0.78 | 55 | 94 | 114 |
|  | Nov. | 12141 | 2707 | 4.34 | 0.95 | 326 | 2321 | 1622 |  | 1966 | 156 | 2.17 | 0.66 | 54 | 104 | 123 |
| 2011 | Feb. | 14229 | 1792 | 3.28 | 0.82 | 235 | 1200 | 1505 |  | 3266 | 200 | 2.21 | 0.71 | 53 | 132 | 189 |
|  | Jun. | 12835 | 1862 | 2.98 | 0.78 | 215 | 1022 | 1116 |  | 4565 | 180 | 1.50 | 0.48 | 42 | 135 | 159 |
|  | Sep. | 14221 | 2652 | 3.70 | 0.90 | 272 | 1937 | 1519 |  | 1995 | 145 | 2.67 | 0.83 | 57 | 113 | 141 |
| 2012 | Jan. | 5834 | 1160 | 3.95 | 0.93 | 289 | 1711 | 1965 |  | 387 | 99 | 3.26 | 0.90 | 86 | 200 | 233 |
|  | Apr. | 11978 | 1344 | 2.27 | 0.62 | 171 | 1315 | 985 |  | 434 | 94 | 3.00 | 0.85 | 73 | 141 | 176 |
|  | Jun. | 13375 | 1579 | 2.67 | 0.71 | 191 | 976 | 1076 |  | 435 | 80 | 2.68 | 0.80 | 64 | 117 | 158 |
|  | Aug. | 9207 | 1389 | 3.01 | 0.78 | 221 | 1250 | 1553 |  | 813 | 139 | 3.05 | 0.84 | 80 | 170 | 180 |
|  | Oct. | 15926 | 2305 | 3.59 | 0.86 | 249 | 1046 | 1168 |  | 576 | 97 | 2.72 | 0.76 | 76 | 149 | 208 |
| Influent sewage | | 3594 | 419 | 2.30 | 0.73 | 144 | 1233 | 1378 |  | 452 | 58 | 1.80 | 0.56 | 45 | 133 | 90 |
| Activated sludge | | 1222 | 257 | 2.85 | 0.74 | 221 | 1187 | 1661 |  | 302 | 44 | 2.73 | 0.87 | 44 | 76 | 73 |
| Total | | 131586 | 15109 |  |  |  |  |  |  | 17635 | 527 |  |  |  |  |  |
| ^a^ Calculation at a sampling depth of 1,000 reads, ^b^ Calculation at a sampling depth of 300 reads | | | | | | | | | | | | | | | | |
